# Complex Posterior Chest Wall Reconstruction Using Bilateral Deep Inferior Epigastric Perforator Flaps After Failed Latissimus Dorsi Free Flap: A Case Report

**DOI:** 10.1002/micr.70284

**Published:** 2026-08-20

**Authors:** Léa Dibiase, Laetitia Ruffenach, Pierre Emmanuel Falcoz, Yann‐Philippe Charles, Pierre Kara, Frédéric Bodin

**Affiliations:** ^1^ Doctor at Plastic Surgery Department Hautepierre Hospital Strasbourg France; ^2^ Doctor at Plastic Surgery Department Haguenau Hospital Haguenau France; ^3^ Head of Cardio‐Thoracic Surgery Department NHC Hospital Strasbourg France; ^4^ Head of Spinal Surgery Department Hautepierre Hospital Strasbourg France; ^5^ Head of Plastic Surgery Department Hautepierre Hospital Strasbourg France

**Keywords:** chest wall reconstruction, chondrosarcoma, DIEP flap, free flap, vein graft

## Abstract

Radical resection of posterior chest wall sarcomas may result in extensive composite defects exposing lung parenchyma, prosthetic material, and spinal instrumentation, while simultaneously exhausting standard pedicled reconstructive options. Salvage reconstruction after free‐flap failure in this setting remains particularly challenging and poorly described. We report the case of a 58‐year‐old patient who underwent en bloc resection of a large posterior chondrosarcoma involving ribs 6–11 and adjacent hemivertebrae, resulting in a massive full‐thickness posterior thoracic defect reconstructed with PTFE mesh and spinal instrumentation. Initial soft‐tissue coverage using a contralateral free latissimus dorsi and serratus anterior flap failed because of early arterial and venous thrombosis. Salvage reconstruction was subsequently performed using bilateral deep inferior epigastric perforator (DIEP) flaps supplied through a Y‐configured saphenous vein graft anastomosed to the thoracodorsal system. The bilateral DIEP flaps provided sufficient surface area and pedicle length extension to achieve tension‐free coverage of the near‐total posterior hemithorax defect. The salvage procedure lasted 10 h in total. Both flaps survived completely without further vascular complications, allowing timely adjuvant radiotherapy. At one‐year follow‐up, the patient demonstrated durable soft‐tissue coverage without reconstruction failure or local recurrence (Figure 3B,C). This case highlights bilateral DIEP flaps combined with Y‐configured vein‐graft extension as a feasible salvage strategy for extensive posterior chest wall defects when conventional latissimus‐based reconstructive options are unavailable or have failed.

## 1 Introduction

Soft‐tissue sarcomas are rare malignancies, accounting for less than 1% of all cancers, and involve the trunk in only a minority of cases (Siegel et al. 2017; Clark et al. 2005). Among primary chest wall tumors, chondrosarcoma is the most frequent histological subtype (Clark et al. 2005). Curative treatment relies on wide en bloc resection with negative margins, which remains the main prognostic factor for local control and survival but often results in extensive composite defects involving ribs, vertebral elements, and overlying soft tissues (Pisters et al. 1996; von Mehren et al. 2014).

Posterior chest wall defects represent a particular reconstructive challenge. They frequently expose lung parenchyma, prosthetic chest wall reconstruction and spinal instrumentation, while local pedicled options may be sacrificed during oncologic resection (Sabolch et al. 2012; Yang et al. 1998; Hoefkens et al. 2016). In this setting, reconstruction must provide durable, well‐vascularized soft‐tissue coverage to protect underlying structures, reduce the risk of infection and permit timely adjuvant radiotherapy when indicated (Sabolch et al. 2012; Yang et al. 1998; Hoefkens et al. 2016).

The ipsilateral latissimus dorsi flap is generally considered the first‐line pedicled option for posterior chest wall reconstruction. When unavailable, a contralateral cross‐over free latissimus dorsi flap, sometimes combined with serratus anterior muscle, is widely regarded as the standard microsurgical solution for large posterior defects (Losken et al. 2004; Bodin et al. 2016; Bosc et al. 2011). However, these strategies may fail or be precluded by previous surgery, pedicle compromise, or early flap thrombosis, leaving very limited reconstructive alternatives.

When conventional latissimus‐based options are exhausted, salvage reconstruction requires creative microsurgical solutions capable of covering extensive defects while accommodating long pedicle distances to suitable recipient vessels. Fasciocutaneous free flaps such as the deep inferior epigastric perforator (DIEP) flap offer large, pliable skin paddles with low donor‐site morbidity, but their use in massive posterior chest wall defects is limited by pedicle length and recipient vessel accessibility.

We report a complex posterior chest wall reconstruction following radical resection of a large chondrosarcoma, in which an initial contralateral free latissimus dorsi plus serratus anterior flap failed early. Definitive salvage was achieved using bilateral free DIEP flaps supplied through a Y‐configured saphenous vein graft to the thoracodorsal system. This case highlights a feasible microsurgical salvage strategy when standard pedicled and latissimus‐based free‐flap options are no longer available.

## 2 Case Report

**FIGURE 1:**
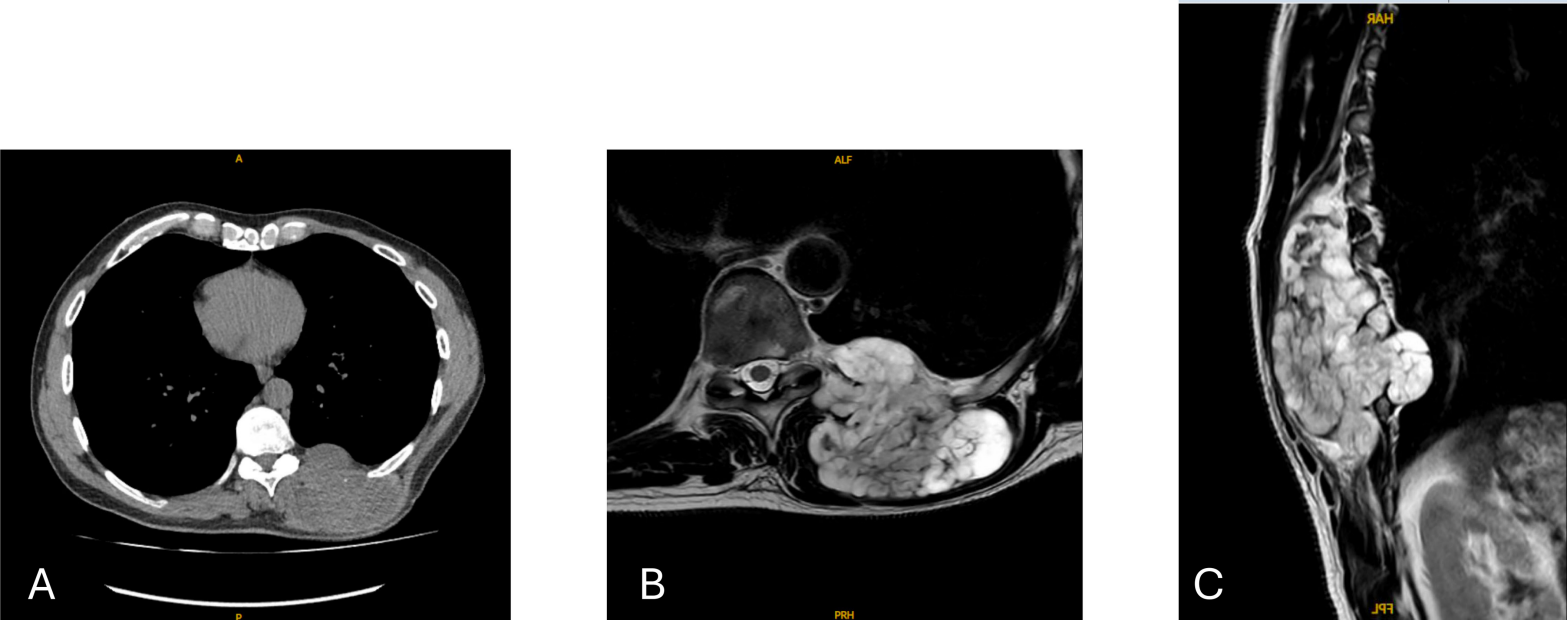
(A) Axial thoracic CT showing a left paravertebral mass involving ribs 9–10 with endothoracic extension. (B) Axial thoracic MRI demonstrating the relationship between the tumor, pleural cavity, and paraspinal muscles. (C) Sagittal thoracic MRI showing craniocaudal extension and contact with vertebral elements.

A 58‐year‐old patient with no significant comorbidities, apart from a remote history of smoking stopped 14 years earlier, presented with a painless left subscapular swelling that had been slowly enlarging for 2 years. Clinical examination revealed a firm, fixed posterior thoracic mass. Thoracic CT imaging demonstrated an 11 × 7 × 9 cm left paravertebral mass centered on the 9th and 10th ribs, with endothoracic extension, calcifications and rib erosion (Figure 1A). Thoracic MRI confirmed extension toward the pleural cavity and paraspinal structures (Figure 1B,C). Biopsy revealed a hyaline cartilaginous tumor consistent with chondrosarcoma. The case was discussed in a multidisciplinary sarcoma board including thoracic, spine and plastic surgeons. A curative en bloc resection was planned, with reconstruction of both skeletal and soft‐tissue components anticipated as a key element of the strategy.

**FIGURE 2:**
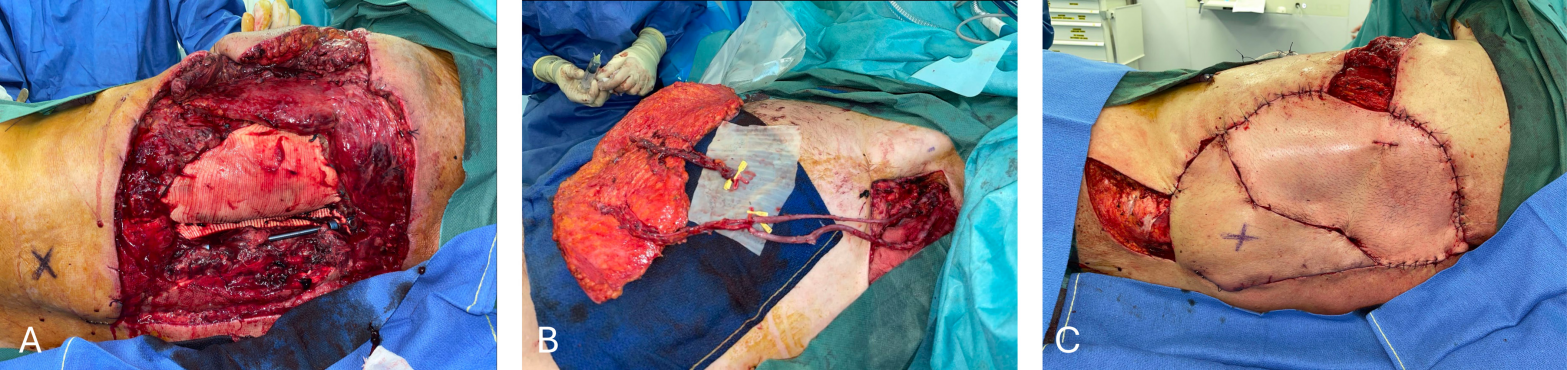
(A) Exposure of the posterior thoracic defect after removal of the failed latissimus flap, showing PTFE chest wall prosthesis and spinal instrumentation. (B) Bilateral DIEP flap preparation: View of the two deep inferior epigastric pedicles before Y‐configuration and saphenous vein graft interposition. (C) Immediate postoperative appearance after inset of the DIEP flaps and coverage of the lower lumbar area with a bilobed perforator flap.

Through a posterolateral approach, the patient underwent lateral and posterior parietectomy from the 6th to the 11th ribs inclusive, associated with hemivertebrectomy of the corresponding levels. Approximately two thirds of the ipsilateral latissimus dorsi muscle were sacrificed as part of the resection. The resulting full‐thickness defect exposed the lung and spine. The internal chest wall was reconstructed with a PTFE (Gore‐Tex) prosthetic patch covering the lung, and the spine was stabilized with posterior instrumentation (Figure 2A).

Soft‐tissue coverage was initially achieved with a combined contralateral free latissimus dorsi flap and serratus anterior flap harvested with a skin paddle. The serratus branch of the thoracodorsal pedicle had been preserved during tumor resection and was used as the recipient pedicle for end‐to‐end anastomosis. At the end of the procedure, the flaps appeared well perfused and covered the entire defect.

On postoperative day two, the patient required urgent re‐exploration for venous and arterial thrombosis proximal to the anastomosis. Repeated thrombosis and revision led to progressive shortening and deterioration of the recipient pedicle. Despite attempts at salvage, flap perfusion remained precarious and the combined latissimus–serratus flap ultimately failed and had to be removed, leaving a massive posterior thoracic defect with exposed lung, prosthetic PTFE mesh and spinal hardware. A postoperative hypercoagulability work‐up did not reveal any underlying thrombophilia or coagulation disorder. Repeated thrombosis was considered mainly related to progressive compromise and shortening of the thoracodorsal recipient pedicle after multiple revisions in a heavily operated field.

Given the lack of local pedicled options and failure of the contralateral latissimus‐based free flap, a salvage strategy using bilateral free DIEP flaps was selected. Preoperative CT angiography had identified dominant medial perforators. The defect size and the shortened thoracodorsal vessels suggested that the native DIEP pedicle length would not be sufficient to reach the recipient vessels without tension, so saphenous vein interposition grafts were planned. The salvage operation was performed 5 days after flap failure by two teams working simultaneously. In our institution, flap compromise is usually managed emergently whenever salvage appears feasible. In the present case, this delay was mainly related to organizational considerations, including completion of complementary imaging studies and coordination of a dedicated operating room with two simultaneous surgical teams for this highly complex reconstructive procedure. One team harvested bilateral great saphenous veins, while the other dissected both DIEP flaps. After closure of the abdominal donor sites, the patient was repositioned in lateral decubitus for posterior inset. A Y‐shaped configuration of the saphenous vein grafts was created to supply both DIEP flaps from the subscapular system (Figure 2B). The left deep inferior epigastric vessels were connected to the subscapular vessels through arterial and venous saphenous vein grafts, yielding an effective pedicle length of about 36 cm. The right DIEP flap was perfused via an end‐to‐end anastomosis between its deep inferior epigastric vessels and the medial branch of the left deep inferior epigastric system. Both flaps were inset to achieve complete coverage of the PTFE mesh, spinal hardware and surrounding soft‐tissue defect (Figure 2C). To address a residual lumbar soft‐tissue defect, a lumbar bilobed perforator flap was harvested based on a perforator identified preoperatively by Doppler and rotated into the defect. Two small marginal areas were allowed to granulate and were later resurfaced with thin split‐thickness skin grafts. The salvage reconstruction procedure lasted 10 h in total, including bilateral DIEP flap harvest, bilateral great saphenous vein harvesting, patient repositioning and construction of the Y‐configured vein graft. Postoperative anticoagulation consisted of standard thromboprophylaxis using low‐molecular‐weight heparin (4000 IU daily) for 3 weeks.

**FIGURE 3:**
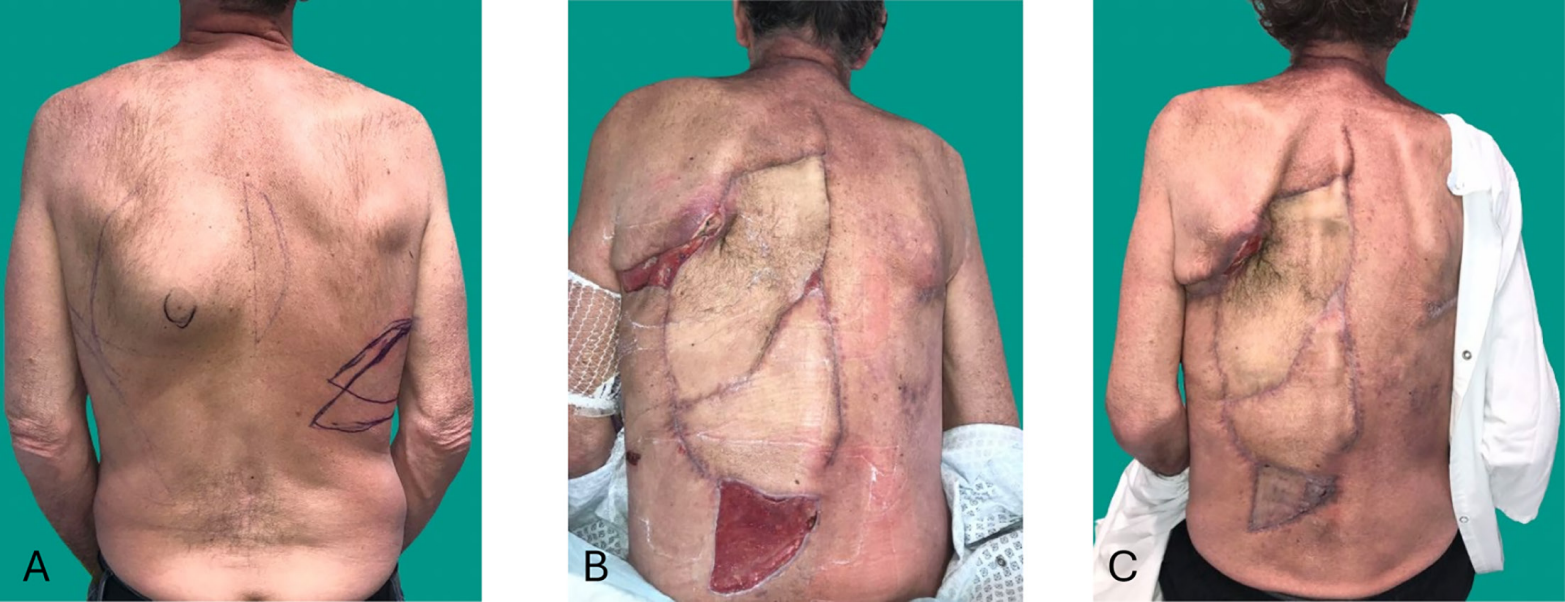
(A) Early postoperative appearance after bilateral DIEP flap inset showing complete coverage of the posterior thoracic defect. (B) One‐year postoperative posterior view demonstrating stable soft‐tissue coverage after adjuvant radiotherapy. (C) Lateral postoperative view at 1 year follow‐up showing durable reconstruction without local recurrence.

The immediate postoperative period required intensive care unit monitoring. Given the risk of deep infection involving lung, spinal cord, PTFE mesh or osteosynthesis material, prolonged broad‐spectrum antibiotic therapy was instituted. Microvascular monitoring revealed no further vascular complications, and both DIEP flaps survived completely. Histopathological analysis confirmed chondrosarcoma with negative margins, allowing adjuvant radiotherapy to the chest wall and paravertebral region. Wound healing was completed within 3 months, and radiotherapy could be delivered without significant delay. At one‐year follow‐up, clinical examination and imaging showed stable soft‐tissue coverage, intact reconstruction and no local recurrence (Figure 3).

## 3 Discussion

Soft‐tissue sarcomas are uncommon and account for less than 1% of all malignancies, with only a minority arising in the trunk (Siegel et al. 2017; Clark et al. 2005). Chondrosarcoma is the most frequent primary malignant tumor of the chest wall (Clark et al. 2005). Curative management relies on wide en bloc resection with negative margins, which is the major prognostic factor for local control and survival (Pisters et al. 1996; von Mehren et al. 2014). However, these margins often translate into large three‐dimensional defects involving bone and soft tissue, especially in the posterior hemithorax.

Posterior chest wall sarcomas are particularly demanding for thoracic surgeons. The need to resect multiple ribs and vertebral elements can result in defects that expose lung, pleural cavity, prosthetic mesh, and spinal instrumentation. Reconstructive failure in this setting may lead to catastrophic complications and may delay adjuvant radiotherapy, which improves local control in high‐risk soft‐tissue sarcomas (Sabolch et al. 2012; Yang et al. 1998; Hoefkens et al. 2016). For thoracic surgeons, early identification of cases in which standard pedicled or latissimus‐based options are unlikely to succeed is crucial, as delayed reconstruction after large posterior resections significantly increases the risk of infection of PTFE prostheses and spinal hardware. In our institution, PTFE mesh remains the standard option for extensive oncologic chest wall reconstruction because of its mechanical reliability and ability to provide durable separation between the thoracic cavity and soft tissues. Biological meshes and acellular dermal matrices may offer advantages regarding tissue integration and infection tolerance, particularly in contaminated fields, but their higher cost and more limited structural rigidity may represent limitations in very large composite defects requiring stable thoracic reconstruction.

Pedicled muscle or musculocutaneous flaps, especially the ipsilateral latissimus dorsi, are usually considered first‐line options for posterior chest wall coverage. When sacrificed or insufficient, a contralateral cross‐over free latissimus dorsi flap, sometimes combined with serratus anterior, is a widely accepted solution because of its proximity, size, and ability to obliterate dead space (Losken et al. 2004; Bodin et al. 2016; Bosc et al. 2011). However, these strategies depend on the availability and quality of recipient vessels, which may be compromised by previous surgery, thrombosis, or irradiation.

Fasciocutaneous free flaps such as the DIEP have emerged as robust alternatives to musculocutaneous flaps. Several series have shown comparable reliability, complication rates, and tolerance to radiotherapy, while reducing donor‐site morbidity and avoiding functional loss of major muscles (Kadle et al. 2019; Othman et al. 2020; Mallett et al. 2021). The extensive perforasome of medial abdominal perforators allows the harvest of a wide and relatively thin skin paddle, which is advantageous for resurfacing extensive thoracic defects (Saint‐Cyr et al. 2009; Lee and Mun 2018). In our case, bilateral DIEP flaps based on medial perforators allowed us to recruit almost the entire abdominal pannus to cover nearly the whole posterior hemithorax.

The main technical limitation to using free flaps for posterior chest wall reconstruction is the distance to suitable recipient vessels. The subscapular/axillary system is often the most practical recipient axis, but aggressive resections and re‐explorations can shorten or damage the thoracodorsal pedicle. In our case, alternative recipient territories such as cervical or intercostal vessels were considered less suitable because of their greater distance from the defect, less favorable orientation, and concerns regarding pedicle reach and vessel caliber. In such situations, interposition vein grafts may be required to restore adequate pedicle length and allow tension‐free anastomosis. Although vein grafts have been associated with higher rates of thrombosis and flap loss in some series (Cheng et al. 2012; Miller et al. 1993), they are typically used in the most challenging cases, where no direct anastomosis is possible and the alternative would be absence of reconstruction or failure of oncologic surgery.

In a large series of free flaps including a subgroup reconstructed with interposition grafts, overall flap survival remained above 90% despite increased complication rates, supporting the use of vein grafts when necessary (Cheng et al. 2012). Our case fits this profile: repeated thrombosis and re‐exploration shortened the thoracodorsal pedicle, and the defect extended over nearly the entire posterior hemithorax. Even a long DIEP pedicle would not have reached the recipient vessels without tension, making a vascular bypass unavoidable.

Bilateral ALT flaps were also considered during salvage planning. However, a single ALT flap was considered insufficient given the size of the defect, while bilateral ALT flaps would likely have required a similar Y‐configured vascular construct with two additional donor sites. Preoperative CT angiography demonstrated highly favorable abdominal perforator anatomy, and bilateral DIEP flaps were estimated to provide a significantly larger skin surface for coverage of the near‐total posterior hemithorax defect.

To our knowledge, reports describing salvage reconstruction of massive posterior chest wall defects using bilateral DIEP flaps supplied through a Y‐configured vein graft after failure of a contralateral latissimus dorsi free flap remain extremely limited.

The Y‐configured saphenous vein graft used in our reconstruction allowed us to perfuse two large DIEP flaps from a single subscapular source and to route the pedicle safely from the axilla to the posterior defect. This strategy provided stable coverage over PTFE mesh and spinal instrumentation and permitted timely adjuvant radiotherapy, which is crucial for local control in chest wall sarcomas.

Our experience suggests several practical points for thoracic surgeons faced with extensive posterior chest wall sarcomas. First, reconstructive planning, including the potential need for vein grafts, should be integrated early into the multidisciplinary discussion. Second, when latissimus‐based options are unavailable or have failed, bilateral DIEP flaps can provide the surface area and pliability required to cover near‐total posterior hemithorax defects. Third, close collaboration with experienced microsurgeons is essential to safely implement complex solutions such as Y‐configured vein‐graft extensions.

This case suggests that bilateral DIEP flaps combined with Y‐configured vein‐graft extension may represent a valuable salvage option for extensive posterior chest wall defects when conventional latissimus‐based strategies are unavailable or have failed.

## Author Contributions

**Léa Dibiase:** data acquisition, manuscript writing, figure creation. **Laetitia Ruffenach:** participation in reconstructive surgery, critical review of the manuscript. **Pierre Emmanuel Falcoz:** thoracic surgery, supervision. **Yann‐Philippe Charles:** spinal surgery, supervision. **Pierre Kara:** major corrections to the manuscript, data acquisition. **Frédéric Bodin:** supervision, major corrections to the manuscript, participation in surgery.

## Funding

The authors have nothing to report.

## Ethics Statement

The authors are accountable for all aspects of the work in ensuring that questions related to the accuracy or integrity of any part of the work are appropriately investigated and resolved. This article does not contain any studies with human participants or animals performed by any of the authors. Written informed consent was obtained from the patient for publication of this case report and all accompanying images.

## Conflicts of Interest

The authors declare no conflicts of interest.

## Data Availability

All relevant data are included in this published article.
